# Nanozyme-Based Colorimetric Assay on a Magnetic Microfluidic Platform for Integrated Detection of TTX

**DOI:** 10.3390/bios16020089

**Published:** 2026-02-01

**Authors:** Chenqi Zhang, Shuo Wu, Fangzhou Zhang, Chang Chen, Jianlong Zhao, Shilun Feng, Bo Liu

**Affiliations:** 1 Xiangfu Laboratory, Jiashan 314100, China; zhangchenqi@xflab.org.cn (C.Z.);; 2 State Key Laboratory of Transducer Technology, Shanghai Institute of Microsystem and Information Technology, Chinese Academy of Sciences, Shanghai 200050, China; 3 Engineering Research Center of Optical Instrument and System, The Ministry of Education, Shanghai Key Laboratory of Modern Optical System, University of Shanghai for Science and Technology, Shanghai 200093, China; 4 School of Microelectronics, Shanghai University, Shanghai 201800, China; 5 Institute of Medical Chip, Ruijin Hospital, Shanghai Jiao Tong University School of Medicine, Shanghai 200025, China; 6 Shanghai Frontier Innovation Research Institute, Shanghai 201108, China

**Keywords:** tetrodotoxin, colorimetric microfluidic immunosensor, magnetic microfluidic chip, aptamer–antibody sandwich assay, on-site rapid detection

## Abstract

Tetrodotoxin (TTX) is a potent marine neurotoxin, necessitating sensitive and user-friendly on-site assays. To address long workflows of traditional immunoassays and limited signal amplification in colorimetric microfluidics, we developed a nanozyme-catalyzed colorimetric magnetic microfluidic immunosensor (Nano-CMI). This platform combines an aptamer–antibody sandwich capture format with catalytic amplification via AuNR@Pt@m-SiO_2_ (APMS) nanozymes on a magnetically actuated microfluidic chip. Magnetic actuation simplifies sample handling and washing, while APMS catalysis enhances sensitivity and visual readout. The Nano-CMI has been used for the detection of TTX samples ranging from 0.2 to 20 ng/mL with a detection limit of 0.2 ng/mL in 10 min, following the linear equation: y = −31.14ln x + 110.15, and the entire “capture-reaction-detection” workflow can be completed within 1 h. With rapid response, minimal hands-on time, and robust performance, this platform offers a practical, high-sensitivity solution for on-site TTX screening in food safety and customs inspection.

## 1. Introduction

Tetrodotoxin (TTX) is a potent marine alkaloid about 1200 times more toxic than cyanide, causing vomiting, respiratory paralysis, and death [1,2,3]. In China, the farmed pufferfish are sold after processing [4], but TTX is heat-stable, and cooking does not remove its toxicity [5]; ingestion of 4–42 μg/kg can cause acute poisoning. With no specific antidote, effective screening from harvest to distribution is essential [3,5,6]. Although most TTX detection research has focused on food safety and marine product monitoring, the toxin also presents serious clinical challenges. Human poisoning often causes rapid neurological symptoms such as paresthesia, paralysis, and potentially fatal respiratory failure, with treatment limited to supportive care (e.g., mechanical ventilation) and no specific antidote available, making timely diagnosis essential. However, current methods, developed mainly for food matrices, often lack the speed, portability, and sensitivity needed for reliable point-of-care quantification in clinical samples like serum, plasma, or urine, particularly in resource-limited settings [3,7].

Liquid chromatography–mass spectrometry/mass spectrometry (LC-MS/MS) is the gold standard for TTX because of its high selectivity and accuracy, but complex workflows, extensive sample preparation, matrix effects, long analysis times, and high instrument costs limit its use for rapid field screening [7,8]. Immunoassays (e.g., ELISA) reduce cost and turnaround but still need laboratory infrastructure and skilled operators, so they do not meet customs or on-site inspection needs [9]. To overcome the limitations of traditional immunoassay, microfluidic immune chips have achieved the advantages of high integration and automation, reducing reagent usage and detection time, and have been well applied in the on-site detection of various analytes [10]. For instance, Ji et al. developed a novel microfluidic immunoassay based on the conjunction with smartphone imaging to realize the rapid detection of okadaic acid in shellfish, achieving on-site detection results within 1 h, with a limit of detection (LOD) of 0.49 ng/mL [11]. Li et al. proposed a fully integrated and high-throughput centrifugal microfluidic system for ultra-multiple point-of-care immunoassay up to 17 samples on a single chip [12]. However, the complex chip structure and the need for professional personnel to use external devices (such as pumps) limit on-site immediate detection, and high costs are not conducive to promotion for resource-limited areas.

Immunodetection of small molecules is challenging because low molecular weight often forces competitive formats with limited sensitivity and dynamic range [13]. Notably, Shkembi et al. first demonstrated that the cage structure of TTX allows formation of an antibody–TTX–aptamer sandwich complex, providing a new avenue for sandwich immunoassays of small molecules [14]. Subsequent research has translated such strategies to lateral flow immunoassay (LFA) platforms, employing gold nanoparticle-labeled antibodies and membrane-bound aptamers to achieve portable rapid test strips with LOD down to 0.3 ng/mL [15]. In addition, signal-on LFAs based on aptamer/antibody recognition have been reported, with detection ranges and LODs of 8–100 ng/mL and 8 ng/mL, respectively [16]. Though these advances highlight the potential of combined antibody and aptamer strategies for small molecule toxin detection, systematic research addressing stability, quantitative accuracy, and highly sensitive, reliable performance in complex matrices remains limited [17,18].

To enhance field detection sensitivity and reliability, nanoscale signal probes and catalytic amplification have emerged as key research directions [19,20]. Quantum dots [21], metal–organic frameworks [22], and electrochemical units [6,23], among other nanomaterials, have been widely adopted for signal enhancement, while nanozymes—nanomaterials with enzyme-like activity—provide robust catalytic amplification [24]. For instance, Ma et al. developed a biomimetic supramolecular nanozyme (He@FF/GA-Apt), achieving a smartphone RGB at LOD of 1.43 ng·mL^−1^ [25]. Additionally, noble-metal nanostructures (e.g., Pt and Pd) exceed traditional enzymes in peroxidase-like activity and can significantly amplify colorimetric or luminescent signals to improve sensitivity [26,27]. Although colorimetric sensors are promising for on-site use, practical field deployment is often limited by procedural complexity; advances in multimodal and composite designs are helping translate nanozyme-enhanced assays to real and complex samples [21,28].

Considering a nanosensor integrated with an on-chip immunoassay, microfluidic immunosensors have shown great promise for on-site high-sensitivity detection [11,29,30]. In a notable application, Cheng et al. integrated porous Au@Pt “nanopompoms” nanozyme-labeled antibodies into a syringe-driven microfluidic chip to achieve rapid and ultrasensitive detection of S. typhi in complex food matrices [31], demonstrating a 91-fold sensitivity boost over HRP-CMI and a 1000-fold improvement over AuNPs-ICA strips. This case illustrates how combining microfluidics with nanomaterial-based signal enhancement not only increases sensitivity but also reduces response time and simplifies signal readout [32].

Magnetic bead (MB)-based magnetic microfluidic systems afford operational advantages, enabling efficient capture, washing, and enrichment through controlled magnetic particle movement between chambers, thereby avoiding errors and contamination from traditional centrifugation and manual washing, and enhancing reproducibility [33,34,35]. The combination of nanozyme signal amplification and magnetic microfluidics enables rapid, sensitive on-site detection of small molecule toxins such as TTX, though robust implementation and systematic validation remain necessary. To our knowledge, this study is the first to realize antibody-aptamer sandwich assay for TTX on a magnetic microfluidic chip, offering a practical route toward customs inspection and food safety screening.

## 2. Materials and Methods

### 2.1. Materials and Instruments

Hexadecyltrimethylammonium bromide (CTAB) was purchased from Amresco (Solon, OH, USA). Potassium tetrachloroplatinate (K_2_PtCl_4_), sodium borohydride (NaBH_4_), chloroauric acid trihydrate (HAuCl_4_·3H_2_O), silver nitrate (AgNO_3_), ascorbic acid (AA), and 3,3′,5,5′-tetramethylbenzidine (TMB) were obtained from Alfa Aesar (Ward Hill, MA, USA). Tetraethyl orthosilicate (TEOS) and ammonium nitrate (NH_4_NO_3_) were purchased from Aladdin (Shanghai, China). Standards of Tetrodotoxin (TTX), saxitoxin (STX), okadaic acid (OA), domoic acid (DA), as well as streptavidin, were purchased from Sigma-Aldrich LLC. (Shanghai, China). PBS, Tween-20, CH_3_OH, H_2_O_2_ (30, wt%), and Bovine Serum Albumin (BSA) were obtained from Sino-pharm Chemical Reagent Co., Ltd. (Shanghai, China). The TTX ELISA detection kit was purchased from Henan Oron Biotechnology Co., Ltd. (Puyang, Henan, China); Tris-HCl (10 mM, pH 9.0) and acetic acid–sodium acetate buffer solution (NaAc-HAc, 0.1 M, pH 4.5) were obtained from Solarbio (Beijing, China). Mouse anti-Tetrodotoxin monoclonal antibody was purchased from Creative Diagnostics (Deltaclon S.L., Madrid, Spain). Ultrapure water used throughout this work was purified by a Milli-Q water purification system. The D3 TTX-binding aptamer with biotin was synthesized from Sangon Biotech Co. Ltd. (Shanghai, China) according to the report from Derosa et al. [14]. The specific sequence is as follows: 5′-biotin-ATACCAGCTTATTCAATTTAATGCGGGGTGAGGCTCAATCAAGGAAAGATATAAGTAAGCAAAAAGGTCAAACAAGGGCGAGATAGTAAGTGCAATCT-3′. A silicone elastomer kit for fabricating a polydimethylsiloxane (PDMS) chip was purchased from Dow Corning (Sylgard 184, Auburn, MI, USA).

### 2.2. Apparatus and Characterization

Optical density or absorbance values were collected by Multi-Mode Microplate Reader (VL0L00D0, Thermo, Waltham, MA, USA) and microvolume spectrophotometer (NanoDrop One, Thermo, Waltham, MA, USA). Zeta potentials are tested with a nanoparticle size zeta potentiometer (Zetasizer Lab, Malvern Panalytical, Malvern, UK). High-angle annular dark-field scanning transmission electron microscopy (HAADF-STEM) was performed on JEM-ARM200F (JEOL, Tokyo, Japan). Colorimetric imaging was acquired using a smartphone. Meanwhile, the intensity of the color signal recorded was processed and analyzed using ImageJ2 (1.54p). Specifically, images were acquired using the rear main camera of a Xiaomi 14 smartphone (Xiaomi Corp., Beijing, China) under a fixed LED illumination (5500 K) at a constant distance, with flash, HDR, filters, and AI enhancement disabled. Camera settings (ISO:1250, aperture: f/2.0, exposure time: 1/100 s, white balance: daylight, and resolution: ~4080 × 3060 pixels) were kept constant for all measurements, and images were analyzed using ImageJ. For each chamber, a fixed-size rectangular region of interest (ROI) was selected within the droplet, and the mean intensity of the red channel was recorded. The mean red intensity of a background region on the chip (without droplet) was subtracted for background correction.

### 2.3. Preparation of the AuNR@Pt@m-SiO_2_ (APMS) Nanorods

Gold nanorods are synthesized by the hydrothermal method; see Supporting Materials for details [36]. AuNR@Pt core–shell nanorods were prepared with slight modifications to a reported procedure [37]. AuNR@Pt@m-SiO_2_ (APMS) nanostructures were prepared through a previous report with some modifications [37]. The 10 mL as-prepared APMS solution was mixed with 50 μL of 0.2 M NaOH and 75 μL of 0.1 M CTAB, and the mixture was kept at 30 °C in a water bath. Three aliquots of TEOS (20 μL each, diluted to 20% in ethanol) were added dropwise at 30-min intervals. The reaction was allowed to proceed for 24 h at 30 °C. The products were collected by centrifugation (12,000 rpm, 10 min) and washed twice. To remove the CTAB template, the precipitate was dispersed in NH_4_NO_3_/ethanol solution (10 mL, 1 mg/mL) at 50 °C in a water bath for 24 h and then centrifuged with ethanol twice (12,000 rpm for 5 min).

### 2.4. Preparation of APMS@mAb Conjugation

In total, 50 μL TTX-mAb (1 mg/mL) was mixed with the as-synthesized APMS solution (50 μL, 5 nM) and diluted to 1 mL with Tris-HCl buffer (10 mM, pH 9), then incubated at 4 °C for at least 24 h [26,38]. Finally, 2% BSA was added as a blocking/storage solution.

### 2.5. Design and Fabrication of Microfluidic Chip

We designed and constructed a highly integrated magnetic-controlled microfluidic chip platform, which consists of six continuous microchambers ingeniously separated by immiscible mineral oil layers to form independent but continuously connected reaction units, achieving a fully automated immunoassay process from sample capture, stepwise washing, nanoenzyme labeling, to signal coloration. Each chip comprises six parallel droplet channels, and each channel contains six circular chambers arranged in a linear array, corresponding to the sequential reaction units for one sample. Thus, up to six samples can be processed in parallel on a single chip (one sample per channel). In this study, all six channels were generally used simultaneously unless otherwise specified. The six channels are separated by PDMS walls and are fluidically isolated from each other; only chambers within the same channel are connected by narrow link channels. Therefore, each channel functions as an independent assay lane. Prior to use, all chambers and link channels were pre-filled with mineral oil. Sample and reagent droplets were then introduced into the inlets using a micropipette at a low dispensing rate, with the pipette tip gently contacting the inlet opening. The mineral oil layer facilitates smooth displacement of air and helps prevent bubble formation, ensuring stable droplets in each chamber.

A key feature of the system is the magnetic actuation module located beneath the chip. In this work, permanent magnets were used to drive the functionalized magnetic beads (MBs) for inter-chamber transfer and in-chamber mixing. The so-called magnetic array is composed of a 3D-printed base and cylindrical permanent magnets of a specific size and grade. The 3D-printed base measures 55 × 45 × 11 mm (length × width × height), and contains circular recesses (grooves) with a depth of 4 mm and a diameter of 3.20 mm for magnet placement. Cylindrical N52 magnets (height 10 mm, diameter 3 mm, nominal surface field ≈ 4700 Gauss) are embedded into these recesses in a precisely designed arrangement. By control of the magnet position beneath each microchamber, MBs can be translated between adjacent chambers and driven to perform directional reciprocating motion within a given chamber, which effectively mimics mechanical stirring. This magnetically driven scheme avoids centrifugation and manual washing steps, thereby reducing operation errors and contamination risks while improving reproducibility.

The microfluidic immunosensor chip is the core component of the detection system [29]. The microfluidic immunosensor chip consists of a PDMS top layer (5 mm) with microfluidic features bonded to a glass substrate (2 mm). Each chip has six droplet channels for TTX, each channel containing six circular reagent chambers in a linear array. Inter-chamber link channels (700 μm wide, 200 μm high) allow MB passage and confine droplets (Figure S1). The PDMS layer was cast from a machined metal mold (Figure S2). Prepolymer and curing agent (10:1 *w*/*w*) were degassed for 30 min, poured, leveled, cured at 80 °C for 2 h, and access holes were punched. The glass was cleaned with ethanol; PDMS and glass were oxygen-plasma-activated, aligned, bonded, and post-baked at 80 °C to strengthen the seal.

### 2.6. Detection for the Nano-CMI

First, a high-concentration TTX standard solution was prepared with a methanol/0.1% acetic acid mixture, and then diluted with PBS to different concentrations. A single assay was run across six magnetically controlled chambers. Each chamber was preloaded with 10 μL mineral oil containing Triton X-100 (1:1000) to prevent evaporation and carryover. In chamber 1, 2 μL MB@Apt (5 mg·mL^−1^) was mixed with 8 μL TTX standards (0–20 ng·mL^−1^ in PBS) and incubated for 15 min, then moved to chamber 2 for a 1 min wash in 0.5% BSA-PBST. BSA-PBST (PBS with 0.05% (*v*/*v*) Tween-20) was prepared fresh. Beads were transferred to chamber 3 containing 10 μL APMS@mAb (2 nM) and incubated for 30 min, followed by two sequential 1 min washes in chambers 4 and 5. Final transfer to chamber 6 (10 μL chromogenic solution: 1 mM TMB, 2 M H_2_O_2_ in 100 mM NaAc-HAc, pH 4.5) allowed color development to be imaged by smartphone RGB within ≤10 min at 25 °C. Beads may be returned to chamber 5 to stop the reaction. To ensure efficient binding kinetics throughout the process, mixing was performed by oscillating a permanent magnet underneath the chip. The magnet was moved in a reciprocating lateral motion at a frequency of approximately 0.5 Hz (30 cycles min^−1^). This dynamic magnetic field induced the continuous dispersion and aggregation of the magnetic bead cluster within the droplet, ensuring thorough sample contact.

## 3. Results

### 3.1. Principle of the Nano-CMI for Capture and Ultrasensitive Detection of TTX

**Scheme 1:**
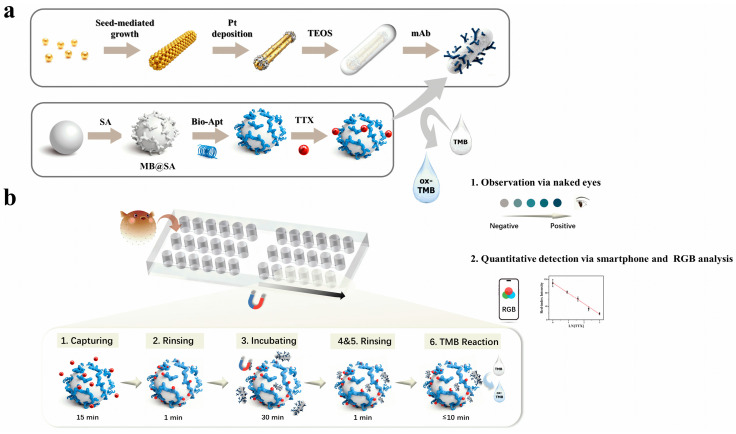
The Nano-CMI for capture and ultrasensitive detection of TTX. (**a**) The design and manufacturing process of conjugates. (**b**) Nanozyme-based colorimetric assay on a magnetic microfluidic platform for integrated detection of TTX.

As illustrated in the mechanism Scheme 1, we developed a six-chamber magnetically controlled microfluidic platform in which immiscible oil phase separate reaction chambers and a fixed magnetic array beneath the chip mediates precise inter-chamber transfer of functionalized magnetic beads, thereby enabling a fully programmatic immunoassay workflow. In the Nano-CMI configuration, atomically dispersed APMS are employed as representative catalytic labels. Owing to their abundant active sites, high peroxidase-like activity, and colloidal stability, APMS provide robust colorimetric signal amplification under detection [37,39]. Although aptamer–antibody sandwich assays have been widely used in bulk formats, their integration into a magnetically actuated droplet microfluidic environment for on-chip biotoxin detection has not been demonstrated.

Herein, TTX aptamer-conjugated magnetic beads introduced into chamber 1 selectively capture the target, and are then magnetically shuttled through sequential wash and reaction droplets to interact with antibody-conjugated APMS in downstream chambers, leading to the formation of highly specific “aptamer-TTX-antibody” sandwich immunocomplexes [14]. After two washes, complexes enter the detection chamber where APMS catalyze TMB/H_2_O_2_ to produce a visible color readout quantifiable by ImageJ. For a typical assay, the approximate durations are as follows: (i) magnetic bead–analyte capture: 15 min; (ii) subsequent incubation: 30 min; (iii) magnetic transfers and washing steps of about 3 min; and (iv) colorimetric development (including image processing time) was less than 10 min, determined by the degree of color development. Thus, the core biochemical workflow on the chip requires approximately 1 h in total, not including initial chip preparation and reagent preloading. In addition, preloaded discrete droplets reduce reagent use and cross-contamination and enable automated, low-volume, multiplexed, rapid, sensitive, and repeatable on-chip TTX detection.

### 3.2. Construction of Magnetron Microfluidic Chip for Immunoassays

**Figure 1:**
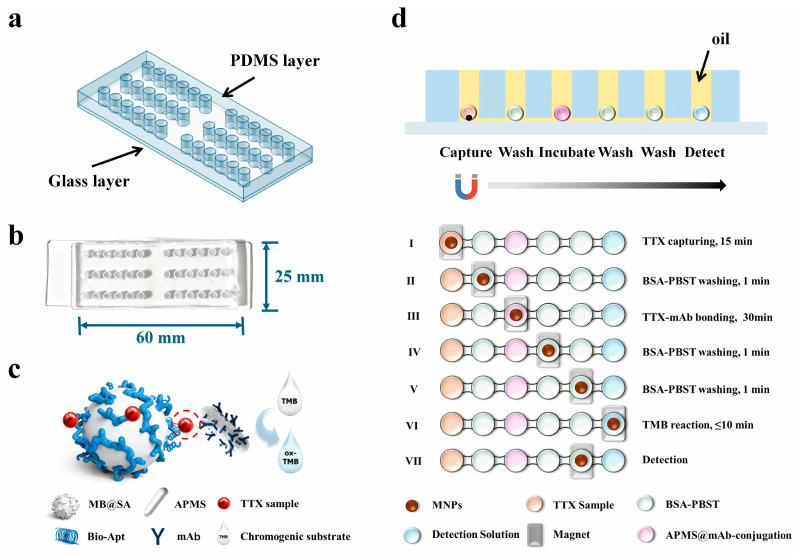
(**a**) A design drawing of the chip. The upper layer is a perforated PDMS channel layer, and the lower layer is a glass supporting layer. (**b**) Picture of the chip used in this work. (**c**) Schematic diagram of the detection principle. (**d**) Schematic diagram of liquid phase reagent filling, the section of the chip, and TTX detection workflow.

**Figure 2:**
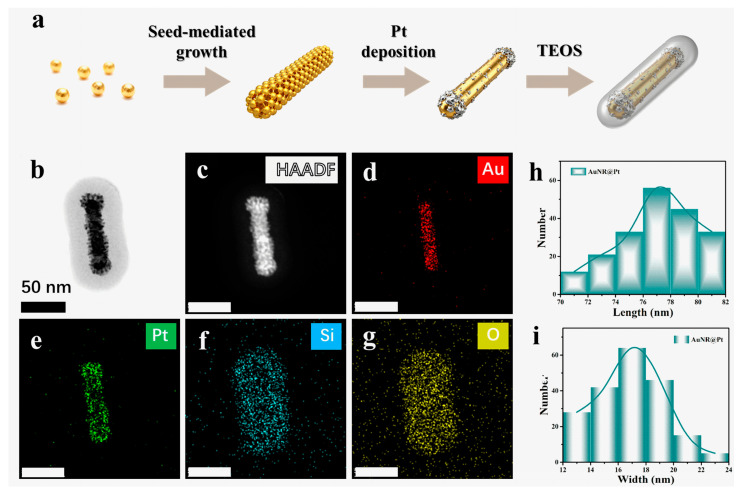
(**a**) Schematic illustration of the hydrothermal synthesis route for AuNR@Pt@SiO_2_ nanorods. (**b**–**g**) HAADF-STEM images of a single AuNR@Pt@SiO_2_ nanorod and EDS elemental maps showing the spatial distributions of Au, Pt, Si, and O, respectively. (**h**,**i**) depict histograms showing the statistical distributions of the length and the width of AuNR@Pt nanorods, respectively.

As depicted in Figure 1a, the microfluidic chip consists of a PDMS layer bonded to a quartz glass substrate. The PDMS layer with microstructures was fabricated using a custom-made metal mold. Briefly, the premixed PDMS was poured into the metal mold and degassed under vacuum to remove air bubbles, followed by curing at 80 °C to form the patterned PDMS layer. Next, both the PDMS surface and the quartz glass substrate were activated by oxygen plasma and then carefully aligned and brought into conformal contact, resulting in irreversible covalent bonding between PDMS and glass and thus the formation of sealed, mechanically robust microfluidic channels. Finally, the assembled chip was placed in an oven at 105 °C overnight to restore the hydrophobicity of the PDMS surface, which is essential for maintaining stable water-in-oil droplet formation within the chambers. The bonded chip, shown in Figure 1b, clearly illustrates the arrangement of reaction chambers and their interconnections. Figure 1c presents MB selectively capturing TTX and forming an immunocomplex through a “aptamer-TTX-antibody”-based sandwich with antibody-functionalized APMS. The operational workflow of the immunosensor is presented in Figure 1d. To isolate droplets from the environment, mineral oil is preloaded into the chip before detection, typically 8–10 μL per chamber, taking care to avoid bubbles. Reagents are then sequentially introduced into the designated chambers (approximately 10 μL each), and the chip is mounted on a magnetic array to perform magnetically controlled transfers. First, a premixed solution of aptamer-functionalized MB and the TTX sample is introduced into chamber 1; under an external magnetic field, MBs are concentrated and moved to capture TTX molecules from the sample, forming MB-TTX complexes. These complexes are then transferred to chamber 2 for washing with 0.5% BSA-PBST (1×, pH 7.4) to remove nonspecific binders. Next, the complexes are moved into chamber 3 to react with immuno-functionalized APMS@mAb, forming “aptamer-TTX-antibody”; after two washeing steps in chambers 4 and 5 for removing unbound proteins on the surface the complexes are transported to the final colorimetric chamber 6, where APMS catalyzes the oxidation of TMB, producing a visible color change. The resulting colorimetric signal is captured by a smartphone and quantitatively analyzed via RGB channel processing in ImageJ, yielding observable and quantitative readouts. Leveraging the APMS nano-catalytic effect shown in Figure 2, Nano-CMI enhances detection sensitivity through catalytic signal amplification while maintaining immuno-specificity, and its magnetically controlled, surface-functionalized architecture confers methodological versatility.

### 3.3. Characterization of APMS Nanorods

The APMS nanorods were prepared via a sequential hydrothermal route, in which preformed gold nanorods were first overgrown with a conformal platinum shell and subsequently encapsulated with an amorphous silica layer (Figure 2a and Figure S3). High-angle annular dark-field STEM and the corresponding energy-dispersive X-ray spectroscopy (EDS) mapping confirm the intended core–shell–shell architecture: the HAADF-STEM contrast differentiates the Au core and Pt shell from the silica overlayer, while the elemental maps for Au, Pt, Si, and O show the expected spatial distributions around the particle (Figure 2b–g). Statistical analysis of the AuNR@Pt cores prior to silica coating yields reproducible size distributions with an average length of 77.3 ± 9.4 nm and an average width of approximately 16.9 ± 1.5 nm (Figure 2h,i), indicating good control over nanorod synthesis and Pt shell formation and supporting the uniformity of the final APMS nanorods [37].

**Figure 3:**
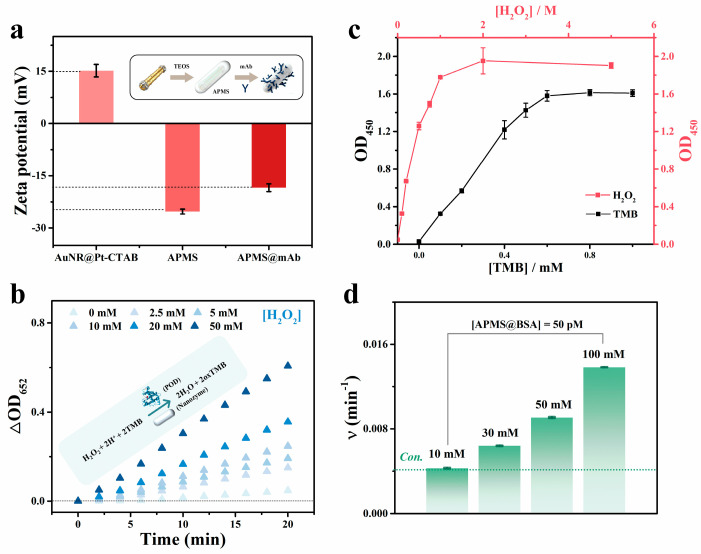
(**a**) Zeta potential profiles of AuNR@Pt nanoparticles, APMS, and APMS@mAb. (**b**) Kinetic analysis of APMS catalyzed reactions as a function of increasing H_2_O_2_ concentration. ([TMB] = 0.5 mM, [NaAc−HAc buffer] = 10 mM, pH 4.5, [particle] = 50 pM). (**c**) Effect of TMB and H_2_O_2_ concentrations on the BSA-coated APMS rate after 10 min. ([NaAc−HAc buffer] = 10 mM, pH 4.5, [particle] = 50 pM) (**d**) Effect of buffer solution concentration on the catalytic rate of BSA−coated APMS particles. ([TMB] = 1 mM, [H_2_O_2_] = 1 M, pH 4.5, [particle] = 50 pM). All experiments start at 25 °C.

Au-core/Pt-shell nanorods with peroxidase-like catalytic activity, as seen in Figure S4. However, when applied to immunoassays, whether the catalytic rate is influenced by the protein corona formed from surface-conjugated antibodies or other carrier proteins remains insufficiently addressed [24,38,39]. Here, BSA-coated APMS@mAb was utilized as the colorimetric signal probe for Nano-CMI, with precise control over its catalytic rate being crucial for signal output. First, as shown in Figure 3a, the zeta potential of APMS shifted markedly after antibody modification, increasing from −25.26 ± 0.70 mV to −18.43 ± 1.11 mV, indicating antibody attachment to the surface. To optimize the reaction conditions, the catalytic performance of the system towards TMB was systematically investigated. At acidic pH with 0.5 mM TMB, increasing H_2_O_2_ markedly enhanced APMS-mediated TMB oxidation, monitored by the OD at 652 nm (OD_652_), demonstrating strong signal amplification (Figure 3b). Although BSA blocking is commonly used to reduce nonspecific adsorption, its effect on APMS peroxidase-like activity remains to be assessed. Optimal chromogenic reagent levels were then determined by monitoring the OD at 450 nm (OD_450_) after a 10-min final reaction (Figure 3c). The results indicated that, with [H_2_O_2_] = 10 mM, the OD_450_ value rose with TMB concentration to a maximum at 1 mM. Conversely, at 1 mM TMB, OD_450_ peaked as H_2_O_2_ reached ~2 M (tested 0–5 M). Furthermore, we investigated the effect of buffer concentration on the catalytic rate of the BSA-coated system. Seen from Figure 3d and Figure S5, at 10 mM NaAc-HAc buffer, BSA had little impact on APMS activity, whereas increasing the NaAc-HAc buffer concentration accelerated reactions for the BSA-coated system. Balancing stability and signal amplification, 100 mM NaAc-HAc buffer was selected as the optimal colorimetric buffer. This tunable chromogenic environment enables precise control of reaction conditions, supporting the sensitivity and reliability of the Nano-CMI assay.

### 3.4. Optimization of Test Conditions

**Figure 4:**
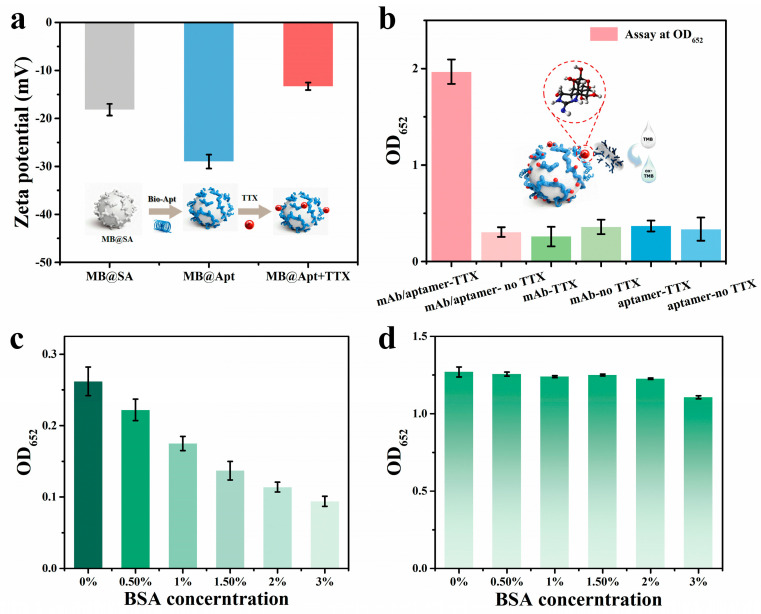
(**a**) Zeta potential changes in the specific MB before and after modification. (**b**) Detection results of antibody−aptamer pairs for the sandwich−conjugation assay. Analysis of blocking efficiency for (**c**) background values and (**d**) signal values.

To demonstrate that the aptamer–antibody sandwich approach is compatible with on-chip detection of TTX, we first performed off-chip immunological validations to assess specificity and feasibility. Zeta-potential measurements were used to monitor surface charge changes in bare MB, aptamer-modified beads, and beads after TTX capture (Figure 4a). The zeta potential of streptavidin beads shifted from −18.20 ± 1.21 mV to −29.0 ± 1.46 mV upon conjugation with biotinylated D3 aptamer, indicating successful surface functionalization. After incubation with TTX for 15 min, the potential increased up to −13.33 ± 0.77 mV, and this significant shift indicates effective TTX capture and the specificity [14,15].

Captured MBs were then mixed with anti-TTX antibody-coated APMS and processed via the MB immunoassay workflow, with OD_652_ measured. Controls containing only antibody or only aptamer showed low OD_652_ (~0.3), whereas samples containing both aptamer and antibody produced OD_652_~2 (Figure 4b), indicating that the concurrent presence of both recognition elements is required to form the “aptamer-TTX-antibody” complexes and incorporate APMS into the detected complex.

Because both MB and APMS are micro/nanoscale particles with high specific surface areas and abundant potential nonspecific adsorption sites [33], suppression of nonspecific binding to reduce false positives is a major concern. After preparing MB@Apt and APMS@mAb, we screened BSA blocking (0.5%, 1%, 1.5%, 2%, and 3%) and measured responses for blank and 1 ng/mL samples (Figure 4c,d). Increasing BSA up to 2% progressively reduced blank color and OD_652_, but higher BSA also suppressed the analyte signal, indicating over-blocking impairs specific binding. Balancing background suppression and signal retention, 2% BSA was chosen as the optimal blocker for subsequent assays [40].

### 3.5. Detection Performances of the Nano-CMI Platform

**Figure 5:**
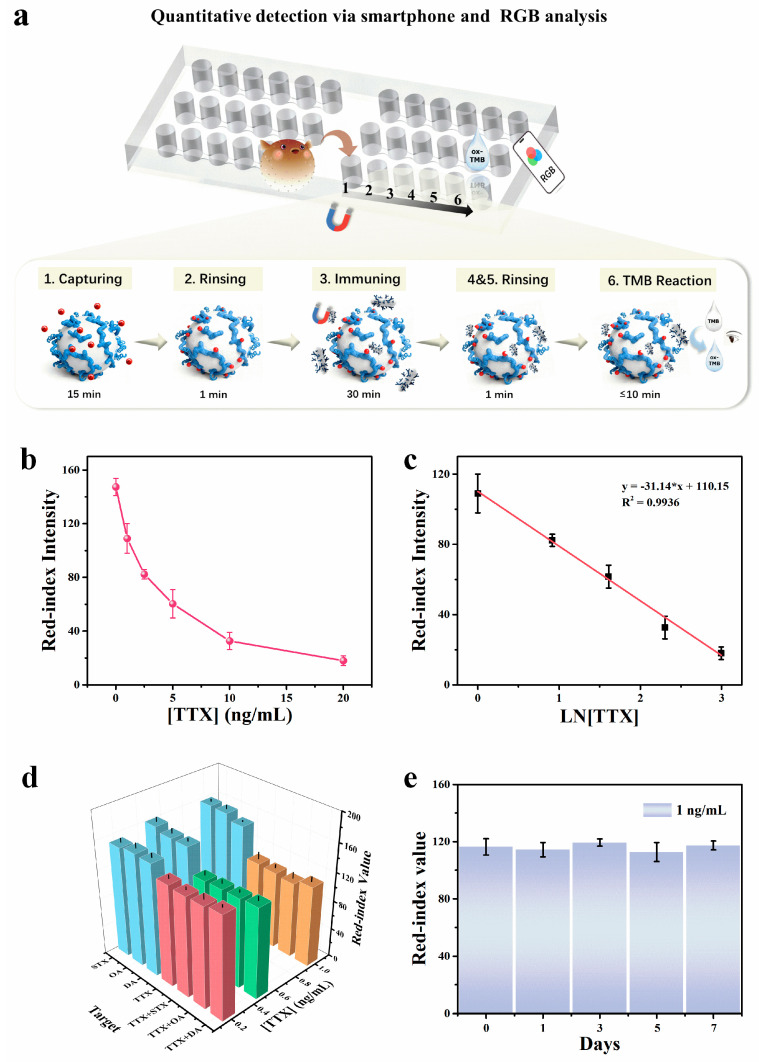
(**a**) Detecting the results of the RGB from Nano-CMI. (**b**) Analytical curve depicting the relationship between TTX concentration and the red-channel intensity of RGB images. (**c**) Calibration curve for the quantitative determination of TTX by immunoassay. (**d**) Selectivity for the detection of TTX under interferences of several common marine biotoxins. (**e**) Evaluation of stability under the assay conditions. All error bars indicate the standard deviation of three parallel experiments. ([TMB] = 1 mM, [H_2_O_2_] = 1 M, pH 4.5, reaction time: 5 min). All experiments started at 25 °C and Data are presented as mean ± standard deviation (*n* = 3).

Differing from commonly used competitive assays for small molecule toxins, this work implements an aptamer–antibody sandwich assay for TTX on a magnetically controlled microfluidic chip [11]. To evaluate the detection performance of the Nano-CMI platform under optimized conditions, parallel measurements were performed for TTX at 0–20 ng/mL, which diluted the mother liquor dispersed in methanol and in 0.1% acetic acid, and finally dissolved in PBS (Figure 5a). As the target concentration increased, the colorimetric reaction intensified. The signal results were analyzed in RGB value by imageJ, and the red index intensity in the image data could better reflect the blue intensity of the oxidized TMB [29], so we here used the red channel value as the quantitative readout. With the colorimetric signal increasing, the red value decreases. As shown in Figure 5b, the red value decreases with increasing TTX concentration. Meanwhile, Figure 5c shows a reverse dependence of the red-index intensity on the logarithm of TTX concentration. The calibration curve is described by I = −31.14 ln c + 110.15 (R^2^ = 0.9936, where c denotes TTX concentration). The limit of detection (LOD) was calculated using the 3SD method. With a visual detection time set to 10 min (typical visual detection windows: 5–10 min), the LOD was determined to be 0.2 ng/mL, and the chosen colorimetric gradients enable clear visual discrimination. This performance is comparable to or exceeds that of previously reported TTX immunoassays (Table 1), highlighting the significant advantages of the Nano-CMI aptamer–antibody sandwich strategy in terms of sensitivity and visual detection capability.

### 3.6. Analysis for the Specificity, Anti-Interference, and Stability

Selectivity and anti-interference were assessed using STX, OA, and DA (10 ng/mL). As shown in Figure 5d, the R-index values obtained from STX, OA, and DA systems were comparable to the blank control, indicating negligible cross-reactivity. In contrast, the TTX system exhibited a distinct signal response, confirming the high specificity of the assay.

To further assess the platform’s reliability in complex environments, interference tests were conducted by spiking 10 ng/mL of interfering toxins into TTX samples. Taking the OA and TTX mixture as an example, when 10 ng/mL OA was co-existing, the R-values for 0.5 ng/mL and 1.0 ng/mL TTX were recorded as 124.33 ± 4.04 and 105.00 ± 9.16, respectively. Similarly, in the mixed system containing 10 ng/mL DA (representing a 1:10 target-to-interferent ratio for 1 ng/mL TTX), the R-value was 112.67 ± 7.51. These results were statistically consistent with the control group of pure 1 ng/mL TTX (R-value = 109.00 ± 10.15), demonstrating that the presence of high-concentration structural analogs or co-occurring toxins does not significantly interfere with the quantitative detection of TTX.

To verify the stability of the developed method, TTX standard samples were still stored in methanol and 0.1% acetic acid, where TTX could retain stability and minimize degradation for at least one week. Repeated detections of TTX were carried out in a week under optimized conditions at a concentration of 1 ng/mL, and as per Figure 5e, the red value at the same reaction time remained consistent, confirming good day-to-day reliability.

Finally, spiked recovery tests were conducted on drinking water to assess practical application. A commercial ELISA kit is used for methodological comparison. The spiked concentrations of TTX are 1, 5, and 10 ng/mL, with six parallel samples measured at each concentration. As presented in Table 2, the spiked recoveries of TTX in water samples range from 91.8 to 105.2%, with a standard deviation (SD) of 1.98 to 7.83%. Using the ELISA kit, the spiked recoveries are 95.6 to 102.2%, with SDs of 3.50 to 5.01%. In contrast, Nano-CMI analysis of drinking water agreed with a commercial ELISA, demonstrating accurate, practical trace TTX detection.

## 4. Discussion

To further evaluate the biofunctionalization of the APMS nanozyme probes and address potential influences of protein coating, we characterized antibody immobilization and catalytic performance post-conjugation. Zeta potential measurements revealed progressive shielding of the negatively charged APMS surface with increasing antibody concentration, reaching saturation at ~50 μg/mL (Supplementary Figure S6). This indicates dense and reproducible antibody loading on the nanorods, which supports consistent multivalent capture capability across batches.

Importantly, comparison of peroxidase-like activity under the final optimized chromogenic conditions (0.1 M NaAc-HAc buffer, pH 4.5, with TMB/H_2_O_2_ substrates) showed that antibody conjugation and subsequent protein layering had a negligible impact on catalytic efficiency of APMS@mAb relative to bare APMS (Supplementary Figure S7). This minimal attenuation by the protein corona, consistent with our earlier kinetic optimizations (Figure 3d and Figure S5), ensures that the intrinsic nanozyme activity is largely preserved in the biological context of the immunoassay. Consequently, the observed signal amplification arises primarily from enhanced local enrichment of nanozyme-labeled detection complexes rather than loss of catalytic function, contributing to both the high sensitivity and excellent batch-to-batch reproducibility of the Nano-CMI platform.

This research demonstrates that Nano-CMI attains sensitivity comparable to or exceeding current signal-amplified TTX immunoassays, with the aptamer-antibody sandwich simplifying workflows [14,15,21,25,41]. Remaining challenges include slow detection due to antigen–antibody kinetics, limiting rapid point-of-care use, and not resolved by the sandwich format [29]. In the current implementation, although the process is automated on-chip, it still entails sequential bead capture, labeling, washing, and color development. To achieve a “true one-step” Nano-CMI assay, future designs should focus on pre-integrating recognition and signaling functions onto a single stable probe. For instance, pre-assembling nanozyme-conjugated nanobodies or aptamer–nanozyme fusion constructs on magnetic beads would allow simultaneous target capture and signal generation immediately upon sample addition. On this specific platform, such a modification would eliminate the need for the ~30 min secondary incubation/labeling step and the associated magnetic transfers, thereby streamlining the on-chip workflow to simply sample loading, a single incubation, and final readout. Faster recognition/signal probes (e.g., high-affinity nanobodies with rapid association rates, or inducible reporters that generate signal directly upon binding without an extra enzymatic amplification step) could be incorporated into the present chip architecture by replacing the current antibody or aptamer–nanozyme conjugates in the bead functionalization step. Because the chip already supports automated magnetic manipulation and multistep washing, these alternative probes would not require major changes to the microfluidic layout, but could shorten incubation time and improve signal-to-background ratios. Direct EM observation of bead–nanozyme complexes remains difficult, because the dynamic nature of the bioconjugation layer and aggregation can mask specific binding events. For this system, correlative microscopy approaches, such as combining fluorescence labeling of the recognition layer with EM imaging of the nanozyme core, would help distinguish specific from nonspecific interactions and provide guidance for adjusting probe spacing and orientation on the bead surface. By (i) redesigning the probes toward pre-assembled, signal-generating capture constructs to approach a one-step assay on this chip, (ii) introducing faster and more compact recognition elements such as nanobodies or inducible reporters into the existing bead functionalization process, and (iii) refining the spatial organization of recognition elements based on structural insights from correlative imaging, theNano-CMII platform could be further developed into a practical tool for rapid toxin and clinical testing [19,30,42,43]. Collectively, with such refinements, Nano-CMI is a promising route to practical, rapid toxin and clinical testing.

In addition to the development of key reagents and raw materials, the development of microfluidic and detection systems is also worth optimizing. In the preliminary work, our group has constructed a droplet microfluidic immunosensing platform based on an automatic magnetron system for CEA detection, combining a programmable magnet array and a displacement platform to realize the automatic manipulation of magnetic nanoparticles between droplets in an oil-phase closed environment [29]. To ensure a long movement stroke, the platform system reserves a large space in the design, so that the overall size of the displacement mechanism is large, which is not conducive to subsequent portability integration. Based on this, this study further developed and expanded into a magnetron microfluidic platform for the detection of TTX, and the platform was designed to be miniaturized. However, its application still has certain limitations. On the one hand, for the detection of targets in complex matrix samples, although the proposed assay demonstrated excellent performance in spiked water, the transition to solid matrices like pufferfish tissue presents additional challenges. Complex matrices may introduce interferents (e.g., lipids and pigments) that could affect the catalytic activity of the nanozymes or cause microfluidic channel clogging. Future work will focus on optimizing on-site extraction protocols (e.g., portable homogenization followed by filtration) and evaluating the stability of TTX in various extraction solvents to ensure the system’s robustness for authentic seafood inspection. Moreover, the current microfluidic chips have not yet integrated upstream sample preparation steps (such as extraction and purification of toxins), although microfluidic modules integrating separation and enrichment units, such as filter membranes, have been reported, and the integration of stable and reliable pretreatment processes into chips for rapid detection in the field is still in the stage of continuous exploration. On the other hand, there is still room for improvement in the overall degree of automation and portability of the existing system. In the follow-up research, we plan to further explore the direction of miniaturized magnetron devices, integrating micro magnetic arrays, motion control modules, uniform light sources, and embedded imaging units (such as smartphones or low-cost cameras), and combining automatic image analysis and data processing algorithms to realize the truly automatic detection process of “sample in and result out” on a portable platform. Such miniaturized magnetron systems are expected to simplify operations and reduce dependence on large-scale experimental equipment, while enabling rapid and multi-index joint immunoassays in the field. By further optimizing the chip structure design, magnetic drive mode, and signal processing strategy, the magnetron microfluidic detection idea has the potential to expand from the current TTX, CEA, and other targets to more clinical and environment-related biomarker detection, thereby promoting magnetron microfluidic immunosensors to practical field detection and point-of-care diagnosis applications.

Beyond food safety and environmental monitoring, the “magnetic droplet-nanozyme” architecture proposed here exhibits significant potential as a versatile platform for broader diagnostic applications, particularly in liquid biopsy. Much like the detection of trace marine toxins, liquid biopsy requires exceptional sensitivity to identify low-abundance biomarkers, such as circulating tumor cells (CTCs) or exosomes, within complex physiological matrices. Addressing this challenge necessitates a robust, integrated “sample-to-answer” workflow [44]. By leveraging the system’s core capabilities, specifically magnetic enrichment for target concentration and droplet compartmentalization for noise suppression, this platform is well-positioned for translation into clinical settings, potentially enabling rapid point-of-care testing in the future.

## 5. Conclusions

Herein, Nano-CMI is a compact microfluidic immunosensor that enables full-process on-site detection of TTX by combining an aptamer–antibody sandwich, nanozyme-mediated signal amplification, and magnetically controlled droplet handling for trace TTX detection. Specifically, based on a six-chamber magnetically controlled microfluidic platform with oil-phase isolation, and combined with a magnetic array beneath the chip, magnetic functionalized beads are programmably transferred between different reaction chambers. Consequently, this design completes integrated operations including capture, washing, reaction, and detection in a fully automated workflow. Meanwhile, the peroxidase-like activity of APMS enables a detection limit of 0.2 ng/mL and a linear range of 0.2–20 ng/mL, thereby demonstrating strong selectivity and stability. This streamlined workflow, which covers capture, reaction, and detection steps within a single chip, thus offers high sensitivity, rapid turnaround, and user-friendly operation, making it suitable for field applications such as customs and food safety monitoring. Furthermore, its modular design supports at least six samples of parallel signal output and multiplexed point-of-care applications. Moving forward, further gains in speed and sensitivity are expected by shortening antigen–antibody binding, optimizing the spatial arrangement of recognition elements, and integrating the magnetic chip into portable devices to completely avoid manual operation.

## Figures and Tables

**Table 1:** Comparison of different detection methods towards TTX.

Detection Method	LOD (ng/mL)	Linear Range (ng/mL)	Time (min)	Reference
Hybrid antibody–aptamer sandwich assay	0.31	0.039–40	150 ^a^	[14]
QD@MOF*Ab based flurescent immunosensor	0.13	0.2–400	15 ^b^	[21]
ZrFe-MOF-based ratiometric fluorescent aptasensor	0.027	0.05–500	55 ^b^	[27]
He@FF/GA-Apt based colorimetric assay	0.61	1.0–40.0	40 ^a^	[25]
AuNP-based lateral flow immunoassay	8	8–100	15 ^b^	[16]
AuNP-based antibody–aptamer sandwich lateral flow assay	0.3	0.31–10	20 ^b^	[15]
APMS colorimetric microfluidic immunosensor	0.15	0.2–20	45 ^a^	This work

“a” and “b” denote the incubation time and determination time of the methods, respectively.

**Table 2:** Detection and recovery results of TTX in real samples of drinking water by the Nano-CMI method and ELISA kits (*n* = 3).

Number		Background (ng/mL)	Added (ng/mL)	Detected (ELISA) (ng/mL)	Spike Recovery (ELISA) (%)	Detected (Nano-CMI) (ng/mL)	Spike Recovery (Nano-CMI) (%)
Drinking water	1	0	1	0.956 ± 0.035	95.6 ± 3.5	0.918 ± 0.030	91.8 ± 3.0
	2	0	5	5.11 ± 0.251	102.6 ± 5.01	5.26 ± 0.392	105.2 ± 7.83
	3	0	10	9.77 ± 0.378	97.7 ± 3.78	9.45 ± 0.198	94.5 ± 1.98

## Data Availability

The original data and contributions presented in this study are included within the article or Supplementary Materials. Data will be made available on request.

## Supplementary Materials

The following supporting information can be downloaded at: https://www.mdpi.com/article/10.3390/bios16020089/s1, Figure S1: Local dimensional parameters of chip mold manufacturing; Figure S2. Fabrication of the metal mold; Figure S3. UV-spectra and STEM images for APMS nanorods; Figure S4. UV-spectra and colorimetric image for catalytic activities of APMS nanozyme; Figure S5. Effects of buffer concentration on the catalytic kinetics of BSA-coated APMS; Figure S6. Zeta potential corresponding to different mAb coating concentrations of APMS@mAb; Figure S7. The change of OD value during reaction kinetics of APMS and APMS@mAb.
