# Supplementary material for: Nanozyme-Based Colorimetric Assay on a Magnetic Microfluidic Platform for Integrated Detection of TTX
Source: Biosensors (Basel). 2026 Feb 1;16(2):89. doi: 10.3390/bios16020089 (PMC12938609; doi:10.3390/bios16020089)
Supplement: Supplementary file 1 [file biosensors-16-00089-s001.zip › biosensors-4109070-supplementary.pdf]

SUPPLEMENTARY INFORMATION

Nanozyme-Based Colorimetric Assay on a Magnetic Microfluidic Platform for Integrated Detection of TTX

Chenqi Zhang [1,2]; Shuo Wu [1,3]; Fangzhou Zhang [1,2]; Chang Chen [4,5]; Jianlong Zhao [1,2,4,6]; Shilun Feng [2] and Bo Liu [1,4]**

1 Xiangfu Laboratory, Jiashan, 314100, China

2 State Key Laboratory of Transducer Technology, Shanghai Institute of Microsystem and Information Technol-ogy, Chinese Academy of Sciences, Shanghai 200050, China

3 Engineering Research Center of Optical Instrument and System, the Ministry of Education, Shanghai Key Laboratory of Modern Optical System, University of Shanghai for Science and Technology, Shanghai, 200093, China

4 School of Microelectronics, Shanghai University, Shanghai 2018004, China

5 Institute of Medical Chip, Ruijin Hospital, Shanghai Jiao Tong University School of Medicine, Shanghai 200025, China

6 Shanghai Frontier Innovation Research Institute, Shanghai 201108, China

*Correspondence: shilun.feng@mail.sim.ac.cn (S.F.); liubo@xflab.org.cn (B.L.); Tel.: +86 18317078225

1. Methods

1.1 Preparation of gold nanorods (AuNR)

First, gold seed solution was prepared by mixing 7.5 mL of 0.1 M CTAB and 100 μL of 24 mM $HAuCl_4$ and diluted to 9.4 mL with deionized water. Then, 0.6 mL of 0.01 M $NaBH_4$ was added under vigorous stirring. After 3 min, the seed solution was kept at 30 °C in a water bath. The growth solution was prepared by mixing 1 mL of 50 mM $HAuCl_4$, 120 μL of 0.1 M $AgNO_3$, 100 mL of 0.1 M CTAB, 2 mL of 0.5 M H_2SO_4, and 800 μL of 0.1 M AA. To initiate the growth of Au NRs, the seed solution (120 μL) was added. After 12 h, the obtained Au NRs were centrifuged twice (12,000 rpm for 5 min) and redispersed in water.

1.2 Preparation of AuNR@Pt nanorods

AuNR@Pt core shell rods were formed by adding K_2PtCl_4 and ascorbic acid to 10 mL of the as prepared AuNR solution (1 nM in 0.2 mM CTAB) to final concentrations of 0.3 mM and 3 mM, respectively, then gently shaken and incubated at 30 °C for 1 h until the solution darkened. After reaction, 10 mL CTAB (0.1 M) was added, the product was centrifuged (9500 rpm, 12 min), washed and redispersed in water at ~5 nM.

1.3 Preparation of the MB@Apt-TTX conjugation

Streptavidin coated magnetic beads (1 μm, 1 mg/mL) were used to immobilize the biotinylated aptamer. Fifty microliters of bead suspension were collected on a magnetic rack, the supernatant removed, washed once with 200 μL PBST (PBS pH 7.4, 0.05% Tween 20) and resuspended in 100 μL PBST with 0.1% BSA. Ten microliters of biotin aptamer (1 μM) were added and incubated at 37 °C, 150 rpm for 30 minutes. Beads were magnetically separated for about one minute, washed three times with 200 μL PBST and finally resuspended in 100 μL PBST. Optional blocking was performed by adding an equal volume of PBST with 1% BSA, incubating 30 minutes at room temperature and washing 1-2 times. For target capture, 10 μL TTX standard (10 ng/mL) was mixed with MB@Apt and incubated at 25 °C, 150 rpm for 15 minutes, then beads were separated, washed and resuspended for zeta potential measurement. Mixing was kept gentle and separation times adjusted as needed.

1.4 Effect of protein coating on catalytic rate of APMS

The buffer environment for the protein-coated system was optimized to ensure both stability and catalytic efficiency. APMS were dispersed in Tris-HCl (10 mM, pH 9), incubated with 300

μg/mL BSA at 37 °C for 2 h, centrifuged, and resuspended in different concentration of HAc buffer (pH 4.5).

2. Figures

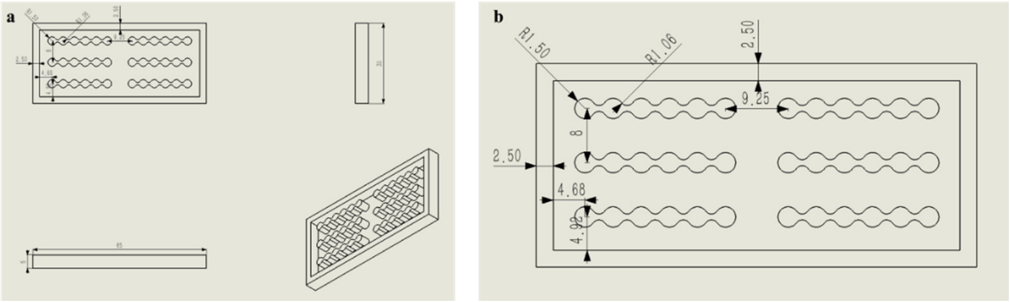

Figure S1. Local dimensional parameters of chip mold manufacturing.

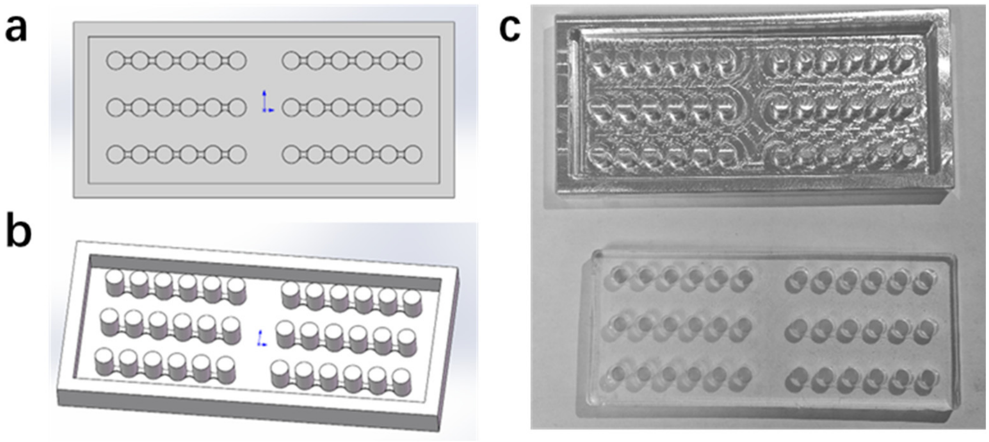

Figure S2. Fabrication of the metal mold. (a) and (b) are the mold's SolidWorks 2D drawing (plan view) and 3D model, respectively; (c) is a photograph of the mold and the PDMS chip.

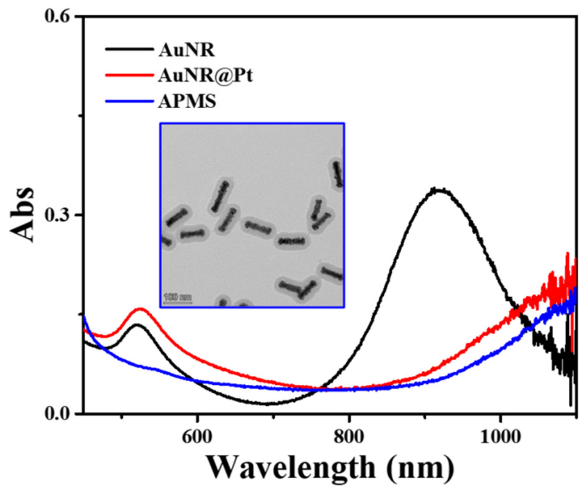

Figure S3. UV-spectra and STEM images for APMS nanorods.

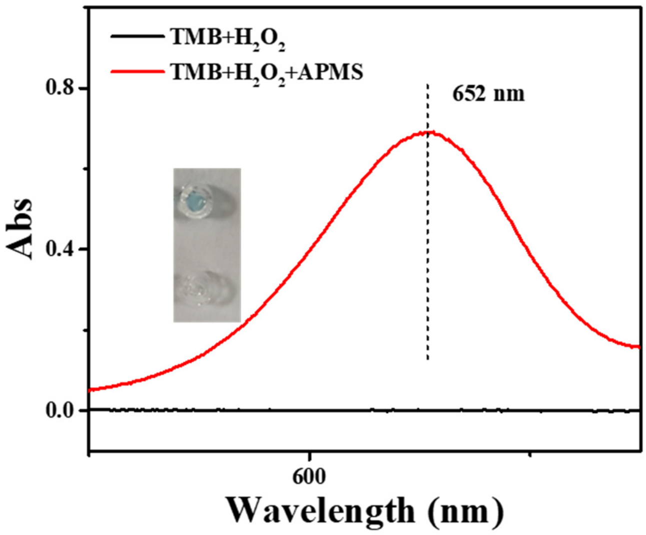

Figure S4. UV-spectra and colorimetric image for catalytic activities of APMS nanozyme.

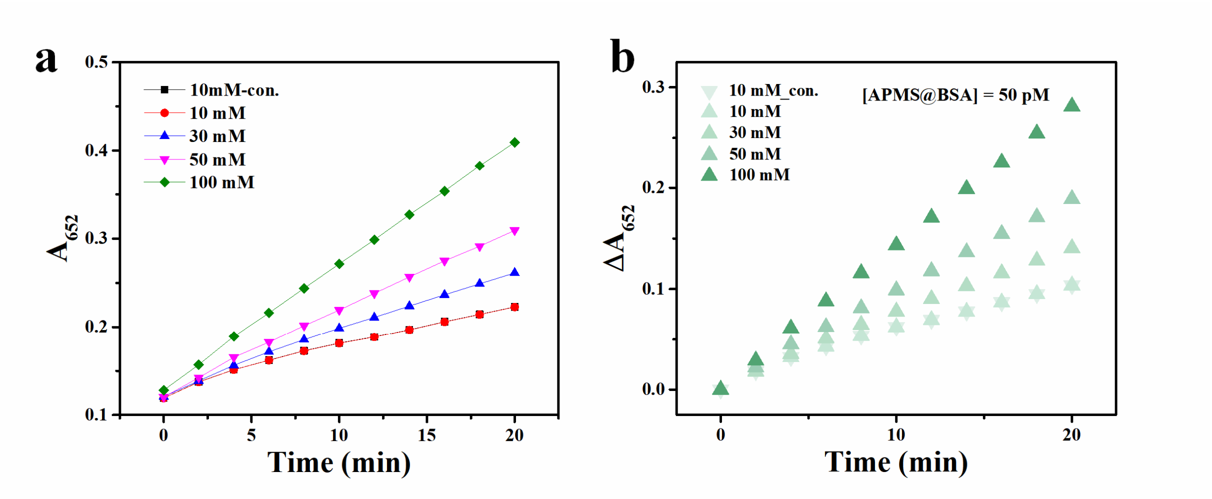

Figure S5. Effects of buffer concentration on the catalytic kinetics of BSA-coated APMS. Panels a and b depict the absorbance (A) and the change in absorbance (ΔA) at 652 nm as the TMB catalytic reaction progresses.

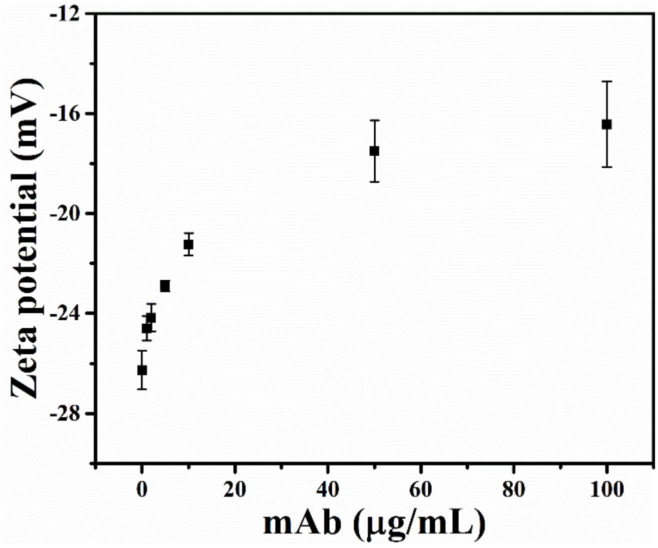

Figure S6. Zeta potential corresponding to different mAb coating concentrations of APMS@mAb.

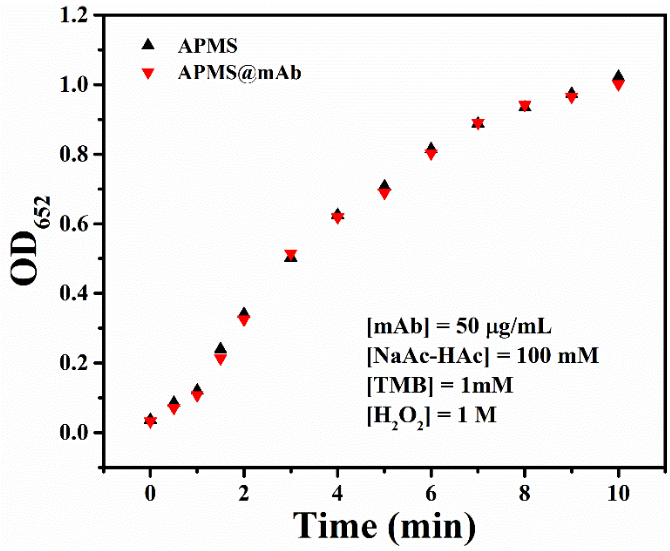

Figure S7. The change of OD value during reaction kinetics of APMS and APMS@mAb.
